# Views of Indian Migrants on Adaptation of Child Oral Health Leaflets: A Qualitative Study

**DOI:** 10.3390/children8010028

**Published:** 2021-01-07

**Authors:** Amit Arora, Roneel Maharaj, Seemagni Naidu, Ritesh Chimoriya, Sameer Bhole, Simone Nash, Charlotte Jones

**Affiliations:** 1 School of Health Sciences, Western Sydney University, Locked Bag 1797, Penrith, NSW 2751, Australia; r.chimoriya@westernsydney.edu.au (R.C.); simonenash_11@outlook.com (S.N.); 2 Translational Health Research Institute, Western Sydney University, Locked Bag 1797, Penrith, NSW 2751, Australia; 3 Discipline of Child and Adolescent Health, Sydney Medical School, Faculty of Medicine and Health, Westmead, NSW 2145, Australia; 4 Oral Health Services, Sydney Local Health District and Sydney Dental Hospital, NSW Health, Surry Hills, NSW 2010, Australia; sameer.bhole@health.nsw.gov.au; 5 Sydney Dental School, Faculty of Medicine and Health, The University of Sydney, Surry Hills, NSW 2010, Australia; rmaharaj87@gmail.com (R.M.); seemagni_n@hotmail.com (S.N.); 6 School of Medicine, Western Sydney University, Locked Bag 1797, Penrith, NSW 2751, Australia; 7 Faculty of Medicine, University of British Columbia, Kelowna, BC V1V 1V7, Canada; charlotte.jones@ubc.ca

**Keywords:** culturally and linguistically diverse, Indian, Hindi, oral health, migrants, Australia

## Abstract

The aim of this study was to gain insight on the views of Hindi-speaking mothers on readily available English language oral health education materials and to evaluate the acceptability of Hindi language adapted versions of these materials. This qualitative study is nested within an ongoing multi-centre birth cohort study in Greater Western Sydney, Australia. Following purposive selection of Hindi-speaking mothers (*n* = 19), a semi-structured interview was conducted. Two English leaflets were mailed to participants prior to the interview. The simplified English and translated Hindi versions of the leaflets were provided at the interview, and the participants were asked to compare and evaluate all three versions. Interviews were audio recorded, and thematic analysis was used to analyse data from interview transcripts. A majority of the participants reported a certain degree of difficulty in reading and comprehending oral health messages in Hindi. Although Hindi translations were accurate, mothers preferred the simplified English as opposed to the Hindi version. Visual illustrations and a simple layout facilitated the understanding of oral health messages. Developers of oral health education leaflets should thoroughly research their prospective user groups, particularly migrant populations, and identify the need for simplified or translated oral health education leaflets.

## 1. Introduction

Early childhood caries (ECC) is defined as “the presence of one or more decayed (non-cavitated or cavitated lesions), missing (due to caries), or filled tooth surfaces in any primary (baby) tooth in a child under the age of six” [1]. According to the Australian National Child Oral Health Study 2012–14, 34.3% of children aged 5–6 years experienced caries in their primary teeth, and over one-quarter had untreated caries in their primary dentition [2]. ECC can have a significant impact on both children and their families [3]. If left untreated, ECC may result in pain, swelling, discomfort, sleepless nights, higher treatment costs, limitations in eating, poor nutrition, reduced growth and development, low self-esteem, frequent absence from school, and distress to the family [3,4]. Numerous biological, behavioural, and socio-demographic risk factors for ECC have been identified, including bacterial infection, predominantly *mutans streptococci*, poor feeding habits such as frequent sugar consumption and night-time bottle-feeding, infrequent toothbrushing with a fluoride toothpaste, poor dental care utilisation, low socio-economic status, cultural beliefs and practices, and low health literacy [5]. Despite the complex interplay of such diverse risk factors, ECC can be prevented through provision of oral health education to families who then adopt healthy practices in early life [6,7]. Oral health education messages are well-established and include brushing with a fluoride toothpaste twice per day, decreasing the frequency and the amount of sugar consumption, and frequent visits to an oral health professional [8].

In health sciences, health education messages are conventionally conveyed through leaflets. However, due to the use of medical jargon and complexity of messages, the usefulness of these resources is highly dependent on the literacy skills of the general public [8,9]. Individuals with low levels of literacy, especially migrants with English as a second language, often have difficulty in understanding and processing the health education messages to make informed decisions about health care [10]. Hence, it is essential to take literacy skills into consideration while developing oral health promotion messages targeted particularly towards the migrant populations.

Health literacy is defined as cognitive and social skills which determine the motivation and the ability of individuals to gain access to, understand, and use information in ways which promote and maintain good health [11]. The Australian Bureau of Statistics (ABS) Adult Literacy and Life Skills Survey (2006) reported that 59% of Australian adults did not have adequate health literacy skills to efficiently and effectively understand and apply health-related information in their daily lives [12]. Moreover, health literacy was poorer in people with lower levels of education, low incomes, low socio-economic status, and among migrants who speak English as a second language [12]. Although the ABS Health Literacy Survey (2018) reported that most Australians found it easy to discuss health concerns and actively engage with their healthcare providers, people who spoke a language other than English at home were less likely to have support for health as compared to those who spoke English at home [13]. Many migrants in Australia come from non-English speaking backgrounds and encounter significant barriers in understanding health literature [9]. Although using one’s native language can be vital for cultural connection and identity, proficiency in English can affect their capacity to access services, pursue higher education, seek employment, and participate in the Australian society [14]. Along with focusing on improvement of health literacy among high-risk populations, interventions targeted at reaching and engaging the population groups with low health literacy should also be prioritised [15].

In the past few decades, there has been a steady rise in Indian migrants in Australia. In 2019, 29.7% of Australia’s population were born overseas, and those born in India represented 2.6% of the total population [16]. India was the third most common birthplace for those born overseas after England and China. There has also been a significant increase in Hindi-speaking population in Australia from 0.5% in 2011 to 0.7% in 2016 [17]. To be specific, the Greater Western Sydney Region in New South Wales (NSW) is of one of the most culturally and linguistically diverse areas of Australia. Here, the largest percentage (3.9%) of the total population were born in India, while 2.1% comprised a Hindi-speaking population in 2016 [18,19]. Moreover, the majority of Indian migrants reside in socioeconomically disadvantaged areas within Greater Western Sydney [20].

In NSW, an abundance of oral health education resources aimed at young children are readily available. Nevertheless, there has been limited research on the cultural appropriateness of these oral health education resources. It is therefore vital to investigate if the oral health education resources address the literacy and the cultural needs of the fast-growing migrant Indian population in Greater Western Sydney. Thus, the aims of this study were to gain insight on the views of Hindi-speaking mothers residing in Sydney on the usefulness of existing English language oral health education leaflets and to evaluate the acceptability of simplified English and translated Hindi versions of the original English leaflets.

## 2. Methods

### 2.1. Selected Original English Oral Health Education Leaflets

With the aim of promoting good oral health in young children, NSW Health has developed various resources, including the two widely distributed leaflets—“NSW Messages for a Healthy Mouth” (L1) [21] and “Teach your baby to drink from a cup” (L2) [22]. “NSW Messages for a Healthy Mouth” integrates five key oral health messages: Eat Well, Drink Well, Clean Well, Play Well, and Stay Well that are further described with supporting evidence and pictures. The leaflet aims to provide comprehensive evidence-based information on oral health that can be utilised by parents and carers for the improvement of overall health of their young children. “Teach your baby to drink from a cup” was developed to promote good oral health by encouraging parents and caregivers to teach their babies to drink from a cup at six months of age. With prominent pictorials, the leaflet provides information on choosing a training cup and teaching the baby to drink from a cup by transitioning from bottle to a cup from 6 to 18 months of age. It also recommends breast milk and other complementary fluids for the baby depending on the child’s age. Both leaflets (L1 and L2) aim to promote good oral health in young children by providing information on risk factors and prevention of ECC.

### 2.2. Development of the Simplified English and Translated Hindi Leaflets

The two original English leaflets (L1 and L2) were modified specifically for Hindi-speaking mothers and carers using standardised principles for linguistic, literacy, and culturally appropriate adaptation of health education resources [23,24,25,26]. For instance, photographs of a conventional Indian family were used, and examples of foods were replaced with those Indian mothers would conventionally feed their baby such as *daal* (lentils), *roti* (bread), *subzi* (vegetables), and *mithai* (sweets). Moreover, simple words were used instead of medical jargon, and the oral health messages were translated into common Hindi terms to ensure that they can be easily comprehended by common Hindi-speaking parents. A multi-disciplinary review team was formed consisting of four project team members (two English-speaking and two Hindi-speaking researchers) and four lay individuals (Hindi-speaking mothers of young children). The team reviewed the original English leaflets (L1 and L2) to identify any potential barriers that would hinder Indian community members’ understanding of the oral health messages. Moreover, they assisted in simplifying the language, reducing medical jargon, and adapting the leaflets based on cultural appropriateness. This led to the development of the simplified English versions of the leaflets (SL1 and SL2).

Subsequently, two Hindi-speaking researchers and two Hindi-speaking lay individuals translated the two simplified English leaflets into Hindi language. As highlighted in literature [27,28], particular attention was given to linguistic and semantic-related issues arising while translating English to Hindi language. Furthermore, the two Hindi-speaking lay individuals back-translated the translated Hindi version of the leaflets to English. This was done to ensure that the oral health education messages in both the adapted versions were consistent in terms of accuracy and concept [26]. Similar to the translation procedure used in other studies [29,30,31], the back-translated versions were compared with the simplified English versions to identify any linguistic and semantic issues and/or translation discrepancies in meaning or terminology. Based on the outcomes at each stage, revisions were made accordingly, leading to the final translated Hindi versions of the leaflets (HL1 and HL2).

### 2.3. Development of the Evaluation Tools

Two distinct semi-structured validated tools [25] were used to evaluate the six leaflets (L1, L2, SL1, SL2, HL1, HL2). The objectives of the evaluation tools were to assess the acceptability and the concept validity of key oral health messages on the basis of cultural norms, literacy, and linguistic skills of the participants and to identify and revise discrepancies in communication, design, and content of simplified English and translated Hindi versions of the leaflets. Each evaluation tool was reviewed by four Indian community members. The reviewers further decided whether the questionnaire could be comprehended by the Indian community and accordingly made minor revisions.

### 2.4. Study Background

This qualitative study is nested within the *Healthy Smiles Healthy Kids* (HSHK) study, a large ongoing birth cohort study in South Western Sydney, Australia that examines the relationship between early childhood feeding patterns, oral health, and obesity [32,33,34]. Mother–infant dyads (*n* = 1035) were recruited by Child and Family Health Nurses at the first post-natal home visit in 2010. As a part of the HSHK study, the two original English leaflets “NSW Messages for a Healthy Mouth” and “Teach your baby to drink from a cup” (L1 and L2) that provide information on oral health of young children were sent by post to the parents before the interview.

### 2.5. Research Design

With the aim of gaining a comprehensive understanding of the preferences of the Hindi-speaking mothers and carers for educative material on oral health, a qualitative research design was used. This allowed in-depth data collection on the participants’ perspectives on all versions of the leaflets as well as simultaneous data collection and data analysis [35].

### 2.6. Sampling Method

Purposive sampling, a non-probability sampling method that incorporates deliberate selection of specific individuals to obtain information-rich data and gain in-depth understanding, was utilised for the study [36]. To further enrich the data quality, maximum variation sampling was used, allowing the capture of varied dimensions of interest and the identification of key patterns [35,36]. Determination of the sample size was based on data saturation. Previous studies have elucidated that 12 to 15 participants is adequate to achieve data saturation, which is the point where little or no new information is being generated that will aid in enhancing the findings of the study [36].

Nineteen Hindi-speaking mothers from the HSHK birth cohort study residing in postcodes classified as “disadvantaged” (2016 Socio-Economic Indexes for Areas (SEIFA) developed by ABS) were chosen for the purpose of this study [37]. To ensure a broader perspective, the following criteria were utilised for the selection of participants:

1. Primiparous or multiparous
2. Any level of education
3. Employed (skilled/unskilled) or unemployed

The selected mothers were invited to participate in the study through a telephone call. An information pack which comprised a participation information statement and a participation consent form, and the two original English oral health education leaflets was mailed to the participants prior to the interview.

### 2.7. In-Depth Semi-Structured Interviews

The simplified English versions (SL1 and SL2) and translated Hindi versions (HL1 and HL2) of both the leaflets were provided to the carers on the day of the interview. Three bilingual researchers (A.A., R.M., and S.N. (Seemagni Naidu)) with prior experience in qualitative interviewing and population oral health conducted in-depth, face to face, one-hour interviews at the participants’ place of residence. Participants were asked to compare and evaluate the content in the two original English leaflets (L1 and L2), followed by the simplified English versions (SL1 and SL2), and finally the translated Hindi versions (HL1 and HL2). Furthermore, the participants were asked to identify the version they believed was the best medium for oral health education. A semi-structured interview framework was selected, allowing for exploration of various related topics of discussion. Derived from our previous qualitative research [8], a semi-structured interview guide (Table 1) was utilised for the interview. During the interview, participants expressed their views, understanding and insights on different versions of the leaflets. All interviews were audio recorded, debriefed, translated from Hindi to English where necessary, and transcribed verbatim.

### 2.8. Evaluation of the Education Materials and Data Analysis

Trained bilingual researchers administered the evaluation tools in the language of choice of participants. Responses to closed-ended questions were expressed in the form of percentages, while answers to open-ended questions were recorded and translated to English where necessary. If the answers given by the participants could not be completely understood by the interviewer, additional time was taken to discuss and clarify.

For qualitative data analysis, five researchers conducted independent debriefing, transcript coding, and interpretation to ensure rigour and credibility. After each interview, the data collected were reviewed, and the key findings were identified. Thematic analysis was used to analyse the data gathered from interview transcripts. Following a line-by-line analysis of all interview transcripts, the principal researcher (A.A.) utilised the NVivo 9 (QSR International, Cambridge, MA, USA) software for first-level coding and identification of common themes. On the other hand, four researchers (R.M., S.N. (Seemagni Naidu), R.C., and S.N. (Simone Nash)) independently performed categorisation, re-categorisation, and condensation of the first-level coding based on similarity and intersection of the responses. Following a thorough review of the transcripts, the concepts were identified. Through an iterative process, the second-level coding along with the corresponding data were reviewed by all five researchers and regrouped into broader themes. Any inconsistencies were resolved through an open discussion between all five researchers to reach a unanimous decision.

### 2.9. Ethics Approval

Ethical approval for this study was acquired from the Human Research Ethics Committee of the former Sydney South West Area Health Service—RPAH Zone (ID number X08-0115), University of Sydney, and Western Sydney University.

## 3. Results

Participants of the study included 19 Hindi-speaking mothers residing in South Western Sydney postcodes classified as “disadvantaged” with children aged 6 months to 3 years. In-depth, face to face interviews were conducted with all participants with a response rate of 100%. Of the 19 mothers, 15 had completed college or university level education, and 14 were employed in either unskilled or skilled occupations. The socio-demographic characteristics of the study participants are outlined in Table 2.

### 3.1. Evaluation of the Simplified English Oral Health Education Leaflets

All 19 participants read the simplified English leaflets (Table 3). Sixteen (84.21%) participants suggested that the simplified English leaflets helped them to understand how to look after their children’s oral health. Thirteen (68.42%) participants indicated that the original leaflets were more difficult to understand compared to the simplified English version. The simplified English leaflets were described as being concise, yet informative, with 18 (94.74%) participants indicating that the leaflets helped them to make better health choices for their child’s oral health.

### 3.2. Evaluation of the Translated Hindi Oral Health Education Leaflets 

Of the 19 participants, three were unable to read the translated Hindi leaflets (Table 4). Of the 16 participants who read the translated Hindi leaflets, 13 (81.25%) expressed that the health education messages written in Hindi were difficult to read compared to the English versions. Nine participants (56.25%) reported that the Hindi leaflets did not help them understand the information about how to look after their child’s teeth any better when compared to the simplified English leaflets. Only six participants (37.50%) found it useful to receive information on child’s oral health in Hindi.

### 3.3. Themes Emerged from the Qualitative Data

From the qualitative data, three major themes emerged: (1) disinclination towards the translated Hindi leaflets, (2) preference for simplified English leaflets, and (3) visual layout facilitates the understanding of health messages.

#### 3.3.1. Theme 1: Disinclination Towards the Translated Hindi Leaflets

Disinclination towards oral health promotion messages written in Hindi language was a recurring theme. The majority of participants expressed difficulty in reading and understanding the translated Hindi-leaflets (HL1 and HL2) as compared to the English versions (L1, L2, SL1, and SL2). The participants indicated that they were acquainted with the oral and the colloquial forms of Hindi language. However, most interviewees provided the insight that English had always been the preferred medium for reading and writing in their day-to-day lives. Hence, there was an overall consensus that, in terms of reading, comprehending, and writing, Hindi language was more demanding than English.

*“I came across some difficulty in reading Hindi because I haven’t read Hindi for a long time. We used Hindi only in our school time and we have done all our work, study and all in English since then, that’s why English is a bit easier to read. It’s funny that we speak Hindi at our home, but we can’t read or write, so it’s a bit more difficult to read than English.”*

Whilst reading in Hindi was difficult, participants indicated that no key information was lost in translation. Some participants suggested that complete translation of English terms was redundant because of their familiarity with English.

*“Many things in India are also in English…like we say ‘fruit’ in India too.”*

Most participants indicated that complexity in comprehension of the oral health messages written in Hindi language itself was a major challenge. Compared to the English versions, the translated Hindi-version leaflets required more time to read as participants were not familiar with reading in Hindi. Interestingly, many participants indicated that the Hindi version would be useful for their parents (child’s grandparents) or those with limited reading skills in English.

*“It took a little bit more time and concentration. I am slower to read Hindi. Sometimes I have to read it by joining the words. If you are studying Hindi from the beginning then it will be easier, but I used to do everything in English so it will be easier for me to read the English one. The Hindi version will be good for my mother (grandmother) as she is used to reading in Hindi.”*

Most of the participants expressed difficulty in understanding Hindi numerals and translated words in the translated Hindi leaflets. Moreover, some participants suggested that colloquial Hindi would be easier to understand than the formal Hindi translation.

*“Sometimes I don’t understand when it is in pure Hindi. Maybe simple Hindi or English is fine. Use words I use in everyday life, not the Hindi I find in my government translations.”*

#### 3.3.2. Theme 2: Preference for Simplified English Leaflets

There was an overall consensus that oral health messages written in simplified English were the favoured medium for oral health education, as this had been the language used during their education, and they had also been required to complete English competency before migrating to Australia. Most participants expressed that the simplified English leaflets were easier to read and understand than the original English leaflets and the translated Hindi-leaflets.

*“It (Hindi) is easy to speak but it is difficult to read and write so that is why simplified English is like crystal clear. I can read it quickly…. I usually read more English because my school was also English medium… We only study (Hindi) in class till fifth class in school, after I picked other language. We also have to pass the English test to migrate to Australia and we read, write, speak and listen in English every day.”*

Preference for simplified English version over original English leaflets for oral health education was a recurring theme. Majority of the participants expressed that the simplified English leaflets were easier to read and comprehend due to the concise content and the clarity in language. Moreover, some participants suggested that simplified English leaflets would be vital for individuals with low English proficiency.

*“The simplified English is excellent. I can read simple words quickly. The short sentences are easy to understand.…it’s very clear. It’s to the point even for someone who has never been to university or even for a foreign second language English, I think that would be good.”*

Most participants highlighted that the simplified English leaflets were culturally sensitive, as they included foods readily used in the local Indian diet and mothers stated that they could relate to the key messages.

*“That it is more relevant for Indian mothers because the examples which are given… it’s the food which Indian mothers feed their baby like Daal, Mithai.”*

#### 3.3.3. Theme 3: Visual Layout Facilitates the Understanding of Health Messages

Interviewees highlighted that that the visual presentation facilitated the understanding of health messages. Most participants conveyed that visual presentation through pictures, Indian foods, Indian families, and a simple layout assisted in their understanding of the relevant key messages. Moreover, some mothers stated that visual illustrations are self-explanatory and can be comprehended without reading the complementary text. That is, in some cases, visual information can assist in elucidating key messages more clearly than words.

*“The picture is so good that anybody can understand, even though he cannot read. I like the thing which you have explained in an image about teeth ring that we have to keep it in fridge. I don’t know about that, so I have not used it properly…”*

The participants’ preference for concise or detailed layout was associated with their experience of being a mother. Detailed educative material was preferred by first time mothers who were keen on gaining a broader perspective. However, mothers with two or more children, or those who worked in child-care, preferred simple concise material to assist with quick recall of prior knowledge.

*“If someone picks and read then there should be enough detail in it, you will not pick one full page and read, if it’s on the notice board in a day-care, it should have basic information.”*

## 4. Discussion

In NSW, although a number of oral health resources are available, there is limited empirical evidence on whether these resources address the oral health literacy needs of the fast-growing Indian migrant population. This study provided insight into the value and the acceptability of simplified English and translated Hindi versions of existing oral health materials that appear to meet the needs of Hindi-speaking mothers in South Western Sydney. A majority of the participants exhibited a certain degree of difficulty in reading and comprehending messages written in Hindi language. There was a consensus that reading and writing in Hindi was more challenging than using the language for verbal communication. The participants preferred messages delivered in simplified English as opposed to Hindi language. Visual representation through pictures and a simple layout were found to further facilitate the understanding of oral health education messages.

In this study, Hindi-speaking mothers showed a disinclination towards translated Hindi leaflets. India is one of the most multilingual countries in the world, where a large number of regional dialects are spoken, including Punjabi, Tamil, and Telegu [38]. It is thought this could have contributed to the participants expressing difficulty in comprehending the Hindi leaflets. English is the auxiliary official language of India. While Hindi is the medium of education in government schools, most private schools provide education in English [39]. Furthermore, higher education in India is mostly delivered in English, and therefore a vast majority of the population prefer to study in private schools from an early age [40]. This could also have been a factor in participants’ disinclination towards Hindi leaflets. A study on Hindi-English bilinguals [41] found an increased difficulty in reading and processing Hindi as compared to English, even with participants who were more fluent in reading in Hindi than English. The authors suggested that the characteristics of Devanagari script can make reading and processing in Hindi more complex and demanding [41].

Simplified English leaflets were the most preferred medium for oral health education over both the original English and the translated Hindi leaflets. It is posited that the aftermath of British post imperialism in India could have been responsible for shaping a generation of learners more confident in regular communication in English [42]. It is also pertinent to note that Hindi-speaking individuals may come from diverse educational backgrounds and may have varying levels of English proficiency, and therefore simplified English may be a preferred method of written communication for Indian migrants. As opposed to the original English leaflets, participants preferred omission of medical jargon in communicating health messages and suggested a greater acceptability of the simplified English version. This reaffirms the findings of similar studies with English-speaking [43], Chinese-speaking [44], Arabic-speaking [8], and Vietnamese-speaking [9] mothers residing in South Western Sydney as participants. Particularly for migrant populations, this study confirms that the provision of simplified English leaflets is more likely to engage target groups with health messages. This finding resonates with a Canadian study that elucidates the significance of simplified health materials for an Indo-Asian community [25]. Cultural appropriateness was found to further enhance the value of simplified English leaflets by making the health messages more relatable, which may have wider implications in the improvement of health disparities [45].

The study participants’ perspectives indicated that visual representation facilitated easy and quick understanding of the key health messages. Pictographic and graphical representation alongside simplified layout was found to have a greater impact than written messages. This finding has been evidenced in the literature [25,46,47] and is supported by experimental psychology and marketing research, which demonstrate that humans have a cognitive preference for picture-based as opposed to text-based information known as the “picture superiority effect” [48]. Similarly, health communication literature [46,47] suggests that images assist in improving patient attention, cognition, recall, and comprehension when literacy levels are low. Particularly in lower health literacy populations, integration of pictorial health information in health communication resources showed a significant improvement in their knowledge and understanding [49]. Teachers of English to Speakers of Other Languages (TESOL) and Teaching English as a Foreign Language (TEFL) classes universally use visual aids for effective and efficient communication and learning [50]. This finding can be particularly useful in English-speaking countries with a high number of migrants.

Engagement, co-creating, and co-designing health leaflets with target audience is vital in the development of oral health resources [51]. The importance of consumer involvement is also evident from similar studies [44,52,53] addressing multi-cultural and multi-linguistic settings. Participant involvement contributes to better comprehension and enhanced literacy, which is the primary objective of the dissemination strategy. Consumer involvement revolves around sharing of lived experiences with developers across all stages of development to ensure that the recommendations made are consistent with their own preferences and values [51]. Moreover, consumer integration in the development phase may help uplift the value of oral health resources to the target audience [8]. Community engagement has also been recognised as an essential tool to address health inequality, which aims at empowering the disadvantaged population and encouraging them to take control of their own health [54]. Particularly in developed countries, individuals with English as a second language are often underrepresented and excluded from health interventions, which may lead to unmet health needs and health disparities [55]. It is therefore imperative for researchers and policy makers to engage consumers in every step to ensure research is translated to policy and practice.

### Strengths and Limitations

This study has several strengths. Firstly, qualitative methodology was utilised to acquire a thorough understanding of the views of Hindi-speaking mothers. The flexible nature of the research design allows for further investigation and simultaneous data collection and analysis [35]. Secondly, this study is nested within the ongoing HSHK birth cohort study, and the 19 Hindi-speaking mothers who were invited to participate did take part for a response rate of 100%. Thirdly, the sample size of 19 participants was adequate to achieve data saturation, which suggests that little or no new information would have been generated with the addition of participants that would aid in enhancing the study findings [36]. Fourthly, the research team constituted of bilingual researchers who provided a cultural insight during the adaptation of the leaflets, the interview process, and the data analysis. Moreover, the evaluation tools developed for the study were reviewed by volunteer Indian community members to ensure acceptability, comprehensiveness, and concept validity of the key oral health messages. Finally, as a result of the HSHK study, a robust partnership has been established with culturally and linguistically diverse communities, which could be beneficial for continuing and further development of culture-specific oral health promotion materials.

This study has a few limitations. Firstly, the participants’ level of comprehension was estimated only by their need for help and personal description of the level of difficulty faced in reading and comprehending the materials compared to any standardised method for estimation. Secondly, the participants’ residency period in Australia and its influence on literacy level was not documented. Thirdly, actual improvement of oral health practices due to the provision of simplified English and translated Hindi leaflets was not assessed. Future studies could measure the differences in immediate and sustained self-reported health behaviours such as frequency of sugar consumption and use of oral care tools to analyse the effectiveness of interventions [56]. Fourthly, as the original leaflets are readily available in NSW, participants may have been familiar with the key messages and also may have acquired assistance to comprehend the content. Nevertheless, the simplified versions provided during the interview were widely accepted by the participants. Finally, the small sample size of Hindi-speaking mothers residing in South Western Sydney limits the external validity of this study, given the broad range of literacy skills amidst other families residing in culturally diverse and disadvantaged regions. However, Hindi-speaking migrants residing in other Western countries may not have significantly divergent views regarding the necessity of appropriate adaptation and translation of health educational materials. Further research into culturally and linguistically diverse communities is essential before developing culture-specific oral health education materials.

## 5. Conclusions

The empirical findings of this study provide a wake-up call to groups entrusted with developing and distributing health education resources to the public. Potential user groups, particularly population groups for whom English is a second language, may have difficulty in reading and understanding the current education materials. At present, oral health leaflets in NSW, Australia are not tailored to meet the needs of migrant mothers of young children. Developing and providing translated leaflets may be a quick alternative but not necessarily the best, as is indicated by the results presented here. The participants demonstrated that oral health literature was best delivered in a simplified English language with culturally appropriate information and minimal medical jargon and was most effective when complimented with generous use of illustrations in a simplified layout. Developing tailored leaflets for individuals with low literacy skills could be beneficial for researchers and policy makers. Participant engagement and information tailored at the level of comprehension of target audience is of utmost importance to encourage compliance and behavioural change. Hence, the developers of oral health education materials should thoroughly research their prospective user groups and identify the indicated need for translated or simplified English oral health education materials.

## Figures and Tables

**Table 1:** Interview guide.

Topics of Discussion for the Interview
1. What are the key messages you got from the leaflet?
2. What made the leaflet easy/difficult to read?
3. Is there anything you were unable to read?
If so, what is that you were unable to read?
4. What made the leaflet easy/difficult to understand?
Is there anything you were unable to understand?
5. If so, what is that you are unable to understand?
6. Did you need help from anyone?
7. Did the leaflet have any missing information?
If so, what was missing?
8. What do you like the most/least about the leaflet?
9. Would you recommend this leaflet for others? Why?
10. Do you have any further suggestions?

**Table 2:** Socio-demographic characteristics of the study participants (*n* = 19).

Characteristic	*n*
Parity	
Primiparous	8
Multiparous	11
Mother’s age (in years)	
20–29	7
30–39	12
Mother’s level of education	
≤year 12	4
College or University	15
Mother’s occupation	
Unemployed	5
Unskilled workers	10
Skilled workers	4

**Table 3:** Simplified English leaflets questionnaire and responses.

Responses to the Questions on Simplified English Leaflets
Did you read the leaflets?
Yes *n* = 19 No *n* = 0
Did you find the simplified leaflets helped you to understand how to look after your child’s oral health?
Yes *n* = 16 (84.21%) No *n* = 3 (15.89%)
What helped you to understand the simplified leaflets?
Simple words
Dot points
Not too much information
Did you find the original leaflets were more difficult to understand compared to the simplified English version?
Yes *n* = 13 (68.42%) No *n* = 6 (31.58%)
Please tell us what part was difficult?
Infant formula
Teething ring
Dental words like sealants
Did you find the simplified leaflets had any missing information compared to the original leaflets?
Yes *n* = 2 (10.53%) No *n* = 17 (89.47%)
What information is specifically missing?
Prefer to have more information
More information could be added such as toothbrushing method
Do the simplified leaflets help you to make better choices for your child’s oral health?
Yes *n* = 18 (94.74%) No *n* = 1 (5.26%)

**Table 4:** Translated Hindi leaflets questionnaire and responses.

Responses to the Questions on Translated Hindi Leaflets
Did you read the leaflets?
Yes *n* = 16 No *n* = 3
Did you find the Hindi leaflets were easier to understand compared to the English versions?
Yes *n* = 3 (18.75%) ^1^ No *n* = 13 (81.25%)
Did you find the Hindi leaflets helped you to understand how to look after children’s teeth compared to the simplified English leaflets?
Yes *n* = 7 (43.75%) No *n* = 9 (56.25%)
Was it useful to receive information on child’s oral health in Hindi?
Yes *n* = 6 (37.50%) No *n* = 10 (62.50%)
What helped you to understand the Hindi leaflets?
Photos
Pictures of Indian foods
Please tell us what part was difficult?
Complex words
Hindi numbers and letters
Can speak Hindi but unable to read well

^1^ Percentages are based on the numbers of participants that read the leaflet (*n* = 16).

## Data Availability

The data presented in this study are not publicly available due to privacy.
